# TB preventive therapy: uptake and time to initiation during implementation of ‘7-1-7’

**DOI:** 10.5588/ijtldopen.24.0101

**Published:** 2024-04-01

**Authors:** A.D. Harries, D. Nair, P. Thekkur, R. Ananthakrishnan, R. Thiagesan, J.M. Chakaya, I. Mbithi, B. Jamil, R. Fatima, M. Khogali, R. Zachariah, S.D. Berger, S. Satyanarayana, A.M.V. Kumar, A.F. Bochner, A. McClelland

**Affiliations:** ^1^ Center for Operational Research, International Union Against Tuberculosis and Lung Disease (The Union), Paris, France;; ^2^ Department of Clinical Research, Faculty of Infectious and Tropical Diseases, London School of Hygiene & Tropical Medicine, London, UK;; ^3^ REACH – Resource Group for Education and Advocacy for Community Health, Chennai, India;; ^4^ Respiratory Society of Kenya, Nairobi,; ^5^ Department of Medicine, Dermatology and Therapeutics, Kenyatta University School of Medicine, Nairobi, Kenya;; ^6^ Department of Medicine, Aga Khan University, Karachi, Pakistan;; ^7^ Common Management Unit (TB, HIV/AIDS & Malaria), Islamabad, Pakistan;; ^8^ Institute of Public Health, College of Medicine and Health Sciences, University of the United Arab Emirates, Al Ain, United Arab Emirates;; ^9^ United Nations Children Fund, United Nations Development Programme, World Bank, Special Programme for Research and Training in Tropical Diseases (TDR), WHO, Geneva, Switzerland;; ^10^ The Union South-East Asia Office, New Delhi, India;; ^11^ Yenepoya Medical College, Yenepoya (deemed University), Mangalore, India;; ^12^ Resolve to Save Lives, New York City, New York, USA

**Keywords:** tuberculosis, TPT, timeliness metrics, household contact screening for TB, the “7-1-7” metric, time to TPT initiation

Dear Editor,

TB preventive therapy (TPT) is an important component of Pillar 1 of the WHO End TB Strategy, which aims to reduce TB incidence by 95% in 2035 compared with 2015.^1^ Household contacts (HHCs) of patients with pulmonary TB (index patients) are at particularly high risk of exposure to TB infection and developing active disease. Because of this, WHO recommends systematic screening of HHCs for TB and initiation of TPT in eligible contacts after active TB has been ruled out,^2^ as this intervention can significantly reduce the risk of TB.^3^ Despite its proven effect, implementation and uptake of TPT in HHCs has been poor. In 2018 at the United Nations High-Level Meeting, world leaders pledged to provide TPT to at least 24 million HHCs by 2022, but only 4.2 million (17.5% of target) received this intervention in the 5-year period.^4^ Not only is TPT uptake sub-optimal, but timely initiation of TPT is also poor, with studies reporting that it can take 2 months or longer for the intervention to be administered.^5,6^ To improve timeliness of HHC screening and TPT initiation, we took inspiration from a new global target, ‘7-1-7’, which has been implemented to improve early detection and rapid control of health threats arising from suspected infectious disease outbreaks and pandemics.^7,8^ We adapted this for the process of HHC management as follows: first ‘7’ – line-listing of HHCs within 7 days of the index TB patient being initiated on treatment; next ‘1’ – line-listed HHCs have symptom screening outcomes ascertained within the next 1 day; second ‘7’ – eligible HHCs start anti-TB treatment or TPT, or a decision is taken to receive no drugs within 7 days of symptom screening.^9^

Over the past 18 months, we have been assessing the feasibility and usefulness of the 7-1-7 metric in HHCs of index patients in four different contexts: the private sector (Chennai, India); the public sector (Chhattisgarh, Bihar; and Odisha, India); the public sector (Sindh Province, Pakistan); and a National TB Programme (Kiambu County, Kenya). The methodology was similar at all the sites and has been described in the preceding papers.^10–12^ One of the important areas we have not yet evaluated is the total number of HHCs initiated on TPT during the implementation of the 7-1-7 metric and the time taken between the index patient starting anti-TB treatment and the HHCs starting TPT. Altogether, there were 1,816 index patients who started anti-TB treatment and a total of 5,166 HHC were line-listed. The cascade from HHCs being line-listed to initiating TPT is shown in Figure 1. Major attrition occurred at two stages of the cascade. The first, and most serious, attrition occurred when 1,548/3,888 (40%) HHCs eligible for further evaluation for TPT after completion of symptom screening failed to attend the health facility. The second occurred when 296/2,317 (13%) HHCs were documented as being ineligible for TPT: this was mostly due to medical officers being reluctant to prescribe treatment, or infrequently due to HHCs having alcohol use disorders and other contraindications to TPT. Altogether, 1,635 HHCs started on TPT. The time taken from initiation of anti-TB treatment in the index patients and initiation of TPT in the 1,635 HHCs is shown in Figure 2. The median time to TPT initiation was 6 days (interquartile range: 3–10). In terms of cumulative initiations, there were 701 (43%) HHCs who initiated TPT within 0–3 days; 1,131 (69%) who initiated within 7 days; 1,480 (91%) who initiated within 14 days; and 1,610 (98%) who initiated within 30 days. The Kaplan–Meier curves, along with median times for TPT initiation, for the four specific contexts are shown in Supplementary Figure S1.

**Figure 1.**
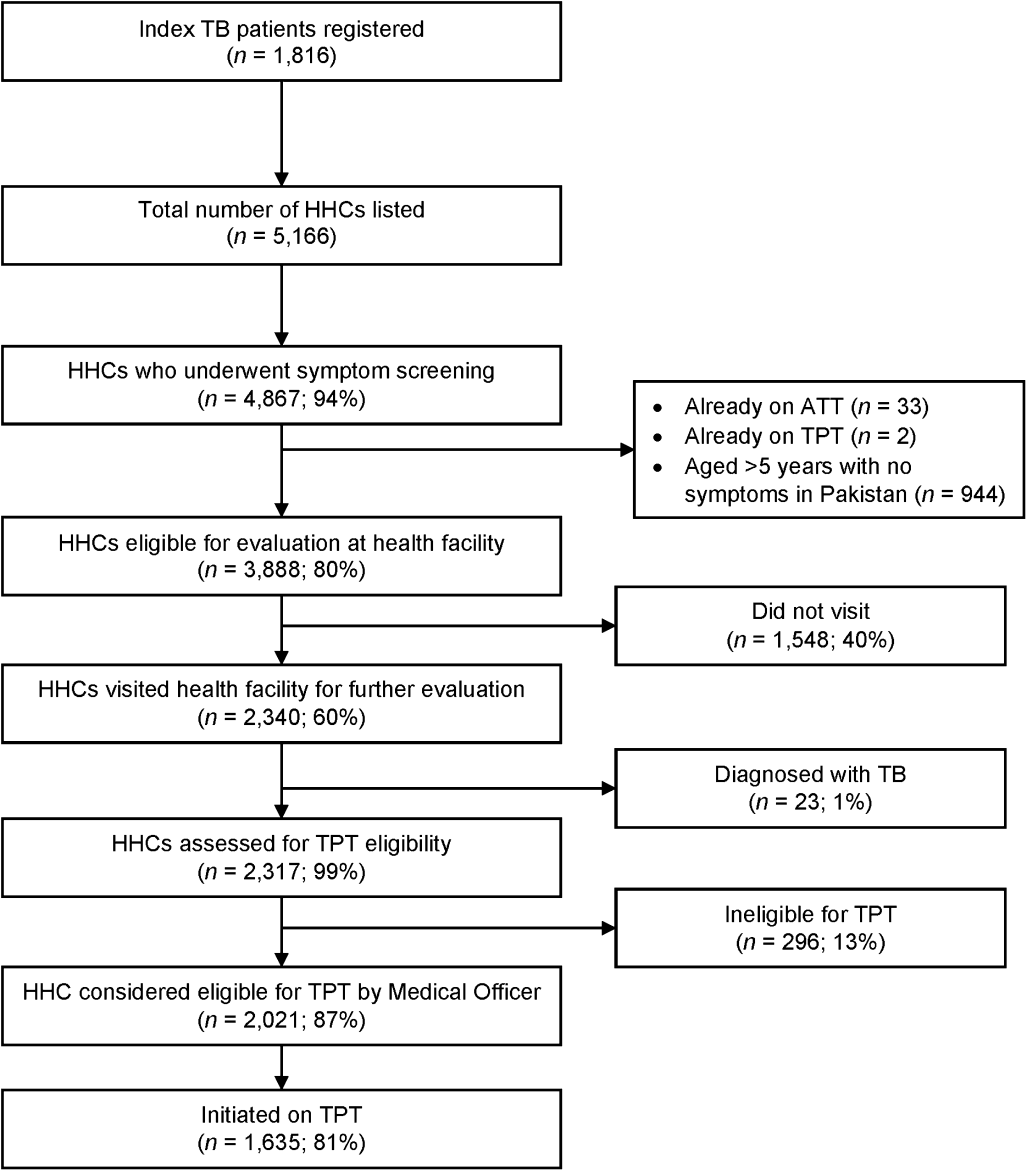
The TPT care cascade among HHCs of pulmonary TB patients in selected sites implementing ‘7-1-7’ timeliness metrics for HHC management in India, Pakistan and Kenya, during 2022–2023. HHC = household contact; ATT = anti-TB treatment; TPT = TB preventive treatment.

**Figure 2.**
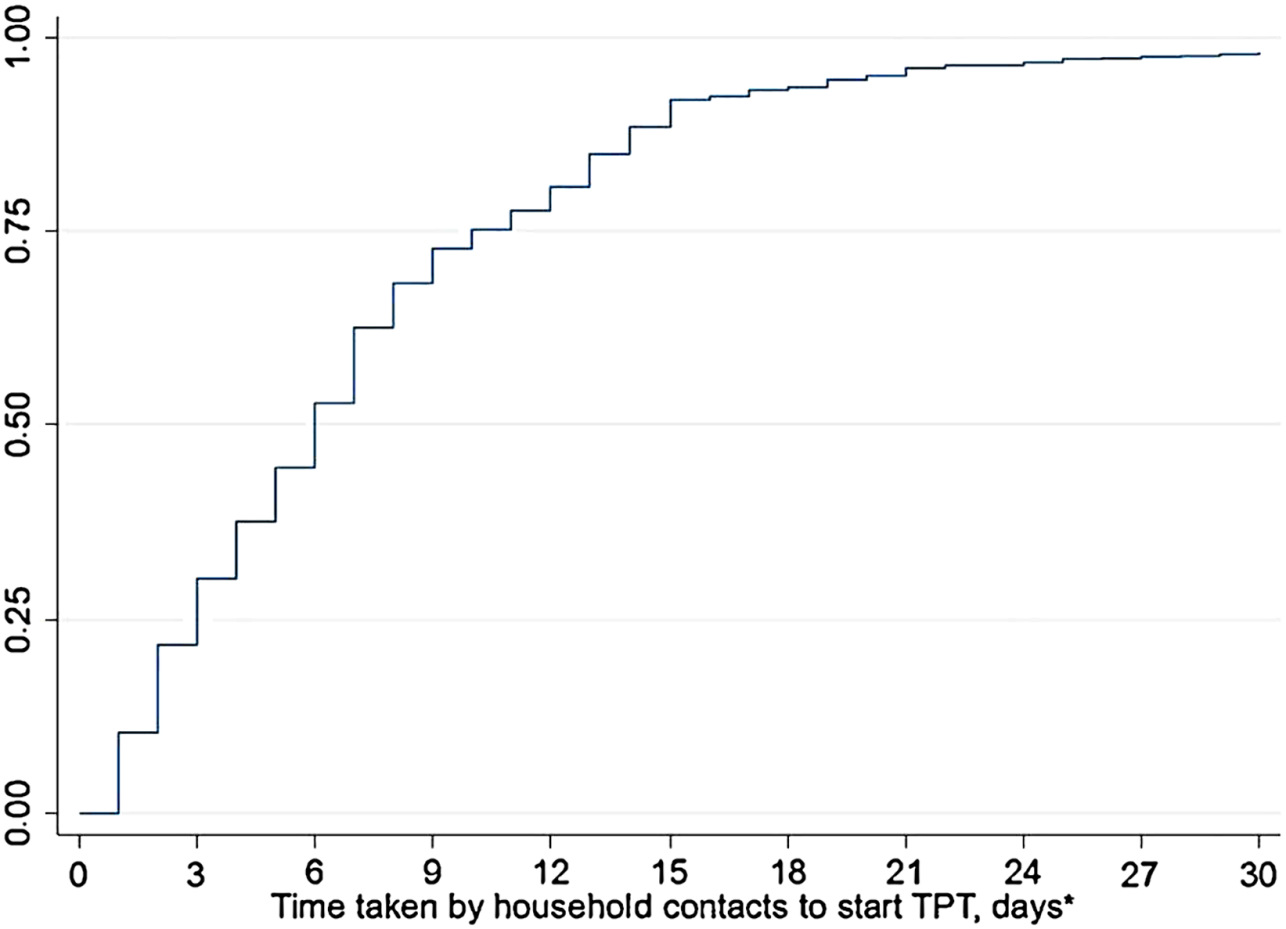
Time to initiation of TPT for 1,635 household contacts measured from the start of anti-TB treatment in index patients with pulmonary TB in selected sites in India, Pakistan and Kenya during 2022–2023. *Overall, there were 25 household contacts initiated on TPT after 30 days. TPT = TB preventive therapy. *Number of days calculated from the day the index patient started anti-TB treatment.

There are two important lessons from these findings. First, the majority of HHCs were initiated on TPT within 30 days of the index patient being treated for TB. Previous evidence indicates a time–response relationship between the treatment of the index TB patient and infection in HHCs, especially in infants and young children, and 30 days appears to be the time from which a significant increase in risk of infection starts to occur.^13^ Thus, it was reassuring that with the 7-1-7 algorithm, over 95% of HHCs commenced TPT within the 30-day time period, with over two-thirds initiating TPT within 7 days. Second, given the speed at which the health workers and HHCs moved to start TPT, it was disappointing that a substantial proportion of HHCs failed to make the journey to the health facility for evaluation, or the medical officer was reluctant to initiate TPT when they did arrive at the health facility. These challenges are not unique to our programme. In many settings, there are recurring themes about costs of travel, loss of daily earnings, direct medical expenses and understandable fears of being diagnosed with TB,^14,15^ all of which make it difficult for apparently healthy HHCs to make the journey to a health facility. Social support to households to cover travel costs and provide reimbursements for loss of daily earnings, and the strengthening of outreach services through the use of mobile vans are some of the initiatives that could help overcome these obstacles. More also needs to be done to educate and persuade health practitioners, index patients and HHCs about the risk of TB in HHCs, the proven benefit of TPT and the notion that prevention is so much better than cure, so that more HHCs can be started on TPT.

The main limitation of this study was the lack of a concurrent control group to assess whether TPT uptake and speed of action was better under the 7-1-7 timeliness metric as compared with having no such target. This should be assessed under operational research conditions. Nevertheless, field staff found that implementation of the 7-1-7 timeliness metric was feasible and helped everyone align on clear goals and objectives. The clear metrics and targets gave structure to the necessary tasks, helped in monitoring the quality of programme implementation and led to improved healthcare worker performance.^10–12^ National TB programmes should seriously consider including timeliness metrics in their work on HHC screening and TPT, and it may be useful in other situations such as time from investigation to diagnosis to treatment for patients with presumptive TB to make this process more efficient and timely.

## Supplementary Material


