# Supplementary material for: TB preventive therapy: uptake and time to initiation during implementation of ‘7-1-7’
Source: IJTLD Open. 2024 Apr 1;1(4):189–91. doi: 10.5588/ijtldopen.24.0101 (PMC11231823; doi:10.5588/ijtldopen.24.0101)
Supplement: Supplementary file 1 [file iutld_ijtld_open_24.0101_supplementarydata1.pdf]

http://dx.doi.org/10.5588/ijtldopen.24.0101

SUPPLEMENTARY DATA

TB preventive therapy: uptake and time to initiation during implementation of '7-1-7'

Supplementary Figure S1: Time to initiation of TPT for household contacts measured from the start of anti-TB treatment in index patients with pulmonary TB in selected sites in India, Pakistan and Kenya during 2022-2023

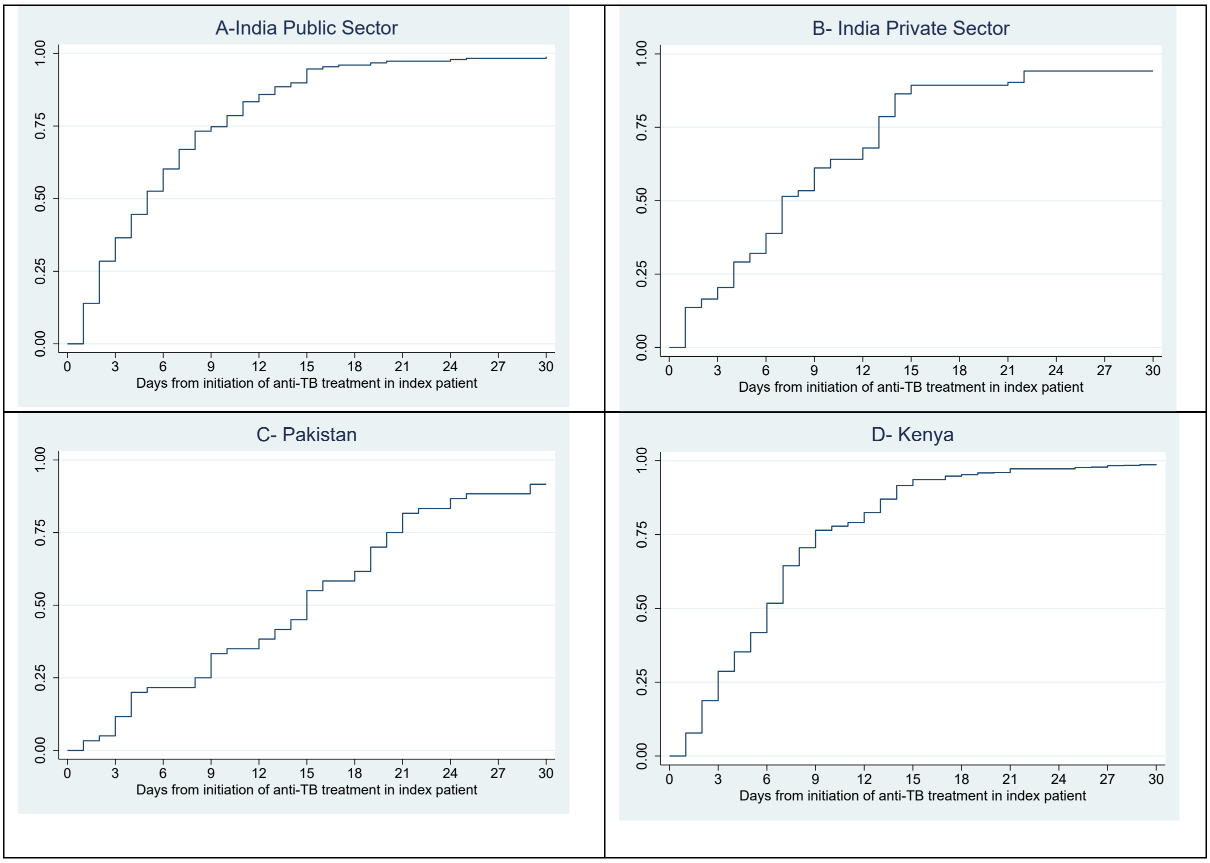

Footnotes: TPT = Tuberculosis Preventive therapy

Median time (interquartile range) in days to TPT initiation:

A-India Public Sector = 5 (2-10) days

B-India Private Sector = 7 (4-13) days

C-Pakistan = 15 (8-20) days

D-Kenya = 6 (3-9) days
